# Complete Remission with Inotuzumab Ozogamicin as Fourth-Line Salvage Therapy in a Child with Relapsed/Refractory Acute Lymphoblastic Leukemia

**DOI:** 10.3390/hematolrep16040056

**Published:** 2024-09-27

**Authors:** Athanasios Tragiannidis, Vassiliki Antari, Eleni Tsotridou, Theodoros Sidiropoulos, Aikaterini Kaisari, Maria Palabougiouki, Timoleon-Achilleas Vyzantiadis, Emmanuel Hatzipantelis, Assimina Galli-Tsinopoulou, Evgenios Goussetis

**Affiliations:** 1 Children & Adolescent Hematology-Oncology Unit, Second Department of Pediatrics, School of Medicine, Aristotle University of Thessaloniki, 54124 Thessaloniki, Greece; antarivaso@gmail.com (V.A.); elentsot@auth.gr (E.T.); tsidirop@hotmail.com (T.S.); palabou.m@gmail.com (M.P.); hatzip@auth.gr (E.H.); agalli@auth.gr (A.G.-T.); 2 Stem Cell Transplant Unit, “Agia Sofia Children’s Hospital”, 11527 Athens, Greece; katerinakaisari@hotmail.com (A.K.); evgoussetis@gmail.com (E.G.); 3 Department of Microbiology, School of Medicine, Aristotle University of Thessaloniki, 54124 Thessaloniki, Greece; tavyz@auth.gr

**Keywords:** acute lymphoblastic leukemia, inotuzumab ozogamicin, childhood

## Abstract

**Background:** Despite the progress achieved regarding survival rates in childhood acute lymphoblastic leukemia (ALL), relapsed or refractory disease still poses a therapeutic challenge. Inotuzumab ozogamicin is a CD22-directed monoclonal antibody conjugated to calicheamicin, which has been approved by the Food and Drug Administration for adults and pediatric patients 1 year and older with relapsed or refractory CD22-positive B-cell precursor acute lymphoblastic leukemia. **Case presentation:** Herein, we present the case of a 23-month-old girl with high-risk B-ALL who experienced very early isolated medullary relapse; following the failure of conventional chemotherapy according to the ALL-IC REL 2016 protocol, she went on to receive the bispecific T-cell engager (BiTE) blinatumomab and subsequently, due to refractory disease, the combination of fludarabine, cytarabine, and the proteasome inhibitor bortezomib without achieving remission. Given the high CD22 expression by the lymphoblasts, off-label use of inotuzumab ozogamicin (InO) was chosen and administrated in a 28-day cycle as a salvage treatment. The minimal residual disease (MRD) was 0.08% on day 28, and InO was continued, thus achieving MRD negativity; the patient successfully underwent an allogeneic stem cell transplantation from a matched family donor. **Conclusions:** Our case highlights the efficacy and safety of InO as a salvage treatment in the setting of relapsed B-ALL refractory not only to conventional chemotherapy but also to novel treatments, such as blinatumomab and bortezomib.

## 1. Introduction

Acute lymphoblastic leukemia (ALL) constitutes the most common malignancy in childhood. Five-year survival rates have increased significantly, exceeding 90%, but relapsed or refractory (r/r) disease still poses a therapeutic challenge [1,2]. Relapse rates have varied from 12% to 19% in recent studies [3,4]. In cases of early medullary relapse, 5-year overall survival (OS) rates remain low, at approximately 30% [4]. Furthermore, achieving remission in order to proceed to hematopoietic stem cell transplantation (HSCT) can be challenging.

Recently novel treatments, including monoclonal antibodies, small molecule drugs, and chimeric antigen receptor T-cell (CAR-T) therapy, have been added to clinicians’ armamentarium. Inotuzumab ozogamicin (InO) is an antibody–drug conjugate comprising a monoclonal antibody targeting CD22 and the cytotoxic agent calicheamicin. Its proposed mechanism of action includes binding to CD22 on the surface of leukemic cells, the internalization of the complex, the fusion of the endosome containing the complex with a lysosome, the release of calicheamicin, the induction of double-stranded DNA breaks, and ultimately cell apoptosis due to irreversible DNA damage [5]. On 6 March 2024, the Food and Drug Administration approved inotuzumab ozogamicin (Besponsa, Pfizer, New York, NY, USA) for pediatric patients 1 year and older with relapsed or refractory CD22-positive B-cell precursor ALL [6]. 

Herein, we report the case of a patient with high-risk disease, very early medullary relapse, and invasive pulmonary aspergillosis during relapse therapy who achieved complete remission with minimal residual disease (MRD) negativity using InO following the failure of salvage conventional chemotherapy, blinatumomab, and bortezomib plus fludarabine and cytarabine and subsequently underwent HSCT.

## 2. Detailed Case Description

A 23-month-old girl was admitted to our department due to fever, leukocytosis, anemia, and thrombocytopenia. The patient’s blood cell count revealed a total white blood cell count of 21.6 × 10^9^/L, a platelet count of 50 × 10^9^/L, and hemoglobin of 71.00 g/L. Notably, bone marrow cytology analysis demonstrated that abnormal lymphocytes comprised 94% of nucleated cells, suggesting a new diagnosis of ALL. Flow cytometry (FC) analysis also confirmed the presence of abnormal lymphocytes expressing CD10, CD19, CD22, CD24, CD34 CD79a, CD105, CD123, and HLA-DR. Karyotype analysis revealed a female hyperdiploid clone with 56 chromosomes (56, ΧX, +X, +4, +6, +8, +14, +17, +18, +21, +22, +mar[18]/46, XX[2]). Translocations t(4;11), t(9;22), t(1;19), and t(12;21) were not detected. The patient also presented with central nervous system (CNS) disease at diagnosis (CNS 3 disease), as indicated by the results of a lumbar puncture. She started induction therapy according to the ALLIC-BFM 2009 [7] and was assigned to the high-risk group due to an FC MRD of 63% on day 15 and 0.95% per protocol criteria. Following reinduction completion, the patient underwent cranial radiotherapy and subsequently started maintenance therapy.

**Figure 1:**
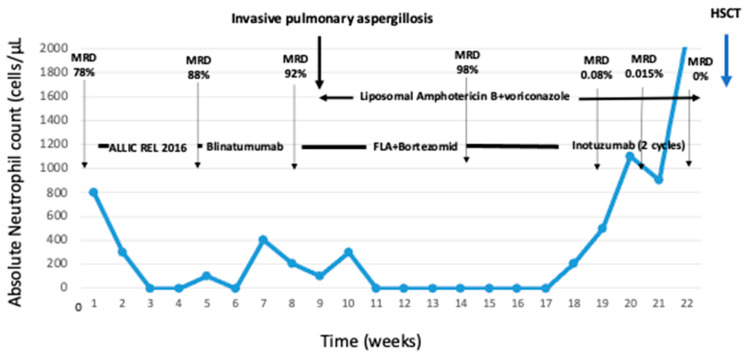
Clinical course of the patients. MRD, minimal residual disease; FLA, fludarabine + cytarabine; HSCT, hematopoietic stem cell transplantation.

However, two months after the initiation of maintenance therapy, the patient presented with leukopenia, neutropenia, and thrombocytopenia. Bone marrow FC analysis showed 78% of abnormal lymphocytes expressing CD19, CD10, CD22, CD9, CD38+partial, and CD58, with the same immunophenotyping panel used upon diagnosis (Figure 1). She underwent chemotherapy according to the ALL-IC REL 2016 protocol [8]. After the completion of the second consolidation block, she underwent a routine FC MRD measurement, which was highly positive (88%). Conventional chemotherapy was discontinued, and the patient was administered the bispecific T-cell engager (BiTE) blinatumomab as a continuous intravenous infusion (15 mg/m^2^). During blinatumomab administration, she developed grade 2 cytokine release syndrome (CRS). Her symptoms were mild and resolved with low doses of dexamethasone and low-flow oxygen supplementation for less than one week. During the third week of blinatumomab infusion, the patient was still agranulocytopenic, and due to low fever, persistent cough, and malaise, a thorax computed tomography (CT) scan and serum galactomannan test were performed. Due to the presence of nodules on the CT scan and positive galactomannan in two consecutive serum samples (>0.5 ng/mL), the patient was diagnosed with probable pulmonary aspergillosis. While on therapy with blinatumomab, the patient was administered combined antifungal treatment with liposomal amphotericin B (3 mg/kg/day iv) and voriconazole (9 mg/kg/bid iv). Due to the infectious complication and the persistence of neutropenia, an FC MRD measurement was performed, which was highly positive (92%). Due to the refractoriness of the disease, despite the fact that immunophenotyping revealed that the blasts were still CD19+, the patient continued combined antifungal treatment for 2 weeks, and without the discontinuation of antifungals, she underwent salvage treatment with the combination of fludarabine, cytarabine (FLA), and the proteasome inhibitor bortezomib. However, the MRD after the completion of the cycle rose to 98%, and the patient showed seropositivity for varicella zoster virus due to bortezomib therapy despite being on prophylactic treatment with acyclovir (Figure 1). 

Finally, given the high CD22 expression by the lymphoblasts, off-label use of InO was selected and administrated in a 28-day cycle with dosing of 1.8 mg/m^2^ divided on days 1 (1 mg/m^2^), 8 (0.5 mg/m^2^), and 15 (0.5 mg/m^2^). InO was administered in combination with liposomal amphotericin B and voriconazole. The MRD was 0.08% on day 28, which led to the decision to proceed to another cycle of InO. InO was well-tolerated, and the patient did not experience CRS, veno-occlusive disease, sinusoidal obstruction syndrome (SOS), or any other adverse events related to its administration. Although the galactomannan values were negative while on InO therapy, the patient continued combined antifungal treatment. Following the completion of the second InO cycle, the MRD was negative. A thorax CT scan showed the amelioration of nodular lesions, with a decrease in their size and number. Liposomal amphotericin B was interrupted, and monotherapy with voriconazole was continued. While in remission, the patient underwent allogeneic HSCT from a matched family donor after a conditioning regimen with treosulfan, fludarabine, and thiotepa. Following HSCT, she experienced another relapse after 5 months and subsequently underwent CAR-T therapy with family donor-derived CAR T-cells. Both HSCT and CAR-T therapy were very well-tolerated, and no long-term post-transplantation complications have been observed. At the last follow-up, the patient was in remission for 12 months. 

## 3. Discussion

Targeted therapies have emerged in the treatment of children and adolescents with hematological malignancies over the past two decades and have revolutionized the field, being used in combination with or after the failure of conventional chemotherapy and/or as a bridging therapy prior to HSCT. Locatelli et al. demonstrated that blinatumomab is superior to a third chemotherapy consolidation block before allogeneic HSCT in patients with a high-risk first relapse in terms of efficacy and safety. Event-free survival (EFS) rates at 22.4 months of follow-up (37% vs. 23%, stratified log-rank test *p*-value < 0.001), as well as MRD remission rates (90% vs. 54%, absolute difference 35.6% (95% CI, 15.6–52.5%)), were significantly higher, and the incidence of serious adverse events was lower (24.1 vs. 43.1%) [9]. However, it must be taken into consideration that eligible patients had M1 or M2 bone marrow status at randomization [10]. Additionally, in the final analysis of the open-label, single-arm RIALTO study on blinatumomab administration in patients with r/r B-ALL, only 21% were refractory to reinduction [9]. Furthermore, in a phase 3 randomized controlled trial by the Children’s Oncology Group (COG) that compared the administration of blinatumomab with consolidation chemotherapy earlier on, i.e., after reinduction in cases of intermediate- or high-risk relapse, the results were also very encouraging, with increased 2-year overall survival (71.3% vs. 58.4%, (hazard ratio for mortality, 0.62 (95% CI, 0.39–0.98); one-sided *p*-value = 0.02)) and MRD remission (75% vs. 32%, absolute difference 43% (95% CI, 31–55%)), but a small subset of patients who received blinatumomab (17%) experienced very early medullary relapse (<18 months from initial diagnosis) [11]. In our case of very early medullary relapse and a rising MRD despite the reinduction and one consolidation block of chemotherapy, there was no response to blinatumomab. Likewise, the use of bortezomib in combination with chemotherapy has rendered very promising results in r/r ALL [12,13]. FLAG (FLA+G-CSF) with bortezomib is particularly appealing due to both the high remission rates (morphological remission of 92% and negative MRD of 61%) and minimal gut toxicity in a heavily pretreated patient population, thus minimizing the risk of bacterial translocation and subsequent sepsis. Of interest, post-bortezomib seropositivity for varicella-zoster virus was detected in our case, as described in the literature [14]. 

CD22 is a B-lineage differentiation antigen that has emerged as an appealing therapeutic target in cases of r/r ALL, as it is expressed on more than 90% of leukemic cells in pediatric patients with relapsed B-ALL but not in cases with MLL rearrangements [15,16]. InO has been approved in adults with r/r B-ALL on the basis of the results of the INO-VATE trial, but data in the pediatric population remain limited [17]. In two retrospective studies on small patient cohorts who received InO on compassionate grounds, very high complete remission (CR) rates were reported (approximately 67% in both studies), with high percentages of MRD negativity among responders [18,19]. It is noteworthy that in a study by Bhojwani et al., the patient population included cases of prior CD22-directed therapy, and the majority of responders achieved CR after only one cycle of InO, similar to our case [18]. The objective response rate (ORR) ranged between 58.3% and 83% in phase 1 and 2 clinical trials of InO as a single agent in patients with r/r B-ALL, with MRD negativity being achieved in the vast majority of responders [20,21,22,23]. Regarding its safety profile, InO was generally well-tolerated, with hematological toxicity, infections, hepatotoxicity, and particularly SOS being the most common and alarming adverse events [20,21,22,23]. SOS in the pediatric population mainly occurs after HSCT. Previous HSCT, conditioning with busulfan or clofarabine, and a shorter time interval between the last dose of InO and HSCT have all been associated with an increased risk of SOS, but there are contradicting data between studies [20,21,22,23]. In our case, hematologic toxicities could not be precisely assessed, as the patient was already experiencing cytopenias, but no hepatic or other toxicity before or after HSCT was observed. 

Our case highlights the efficacy and safety of InO as a salvage treatment in the setting of relapsed B-ALL refractory not only to conventional chemotherapy but also to novel treatments, such as blinatumomab and bortezomib. 

## Data Availability

The original contributions presented in the study are included in the article, further inquiries can be directed to the corresponding author.

## References

[1] Hunger S.P., Lu X., Devidas M., Camitta B.M., Gaynon P.S., Winick N.J., Reaman G.H., Carroll W.L. (2012). Improved survival for children and adolescents with acute lymphoblastic leukemia between 1990 and 2005: A report from the children’s oncology group. J. Clin. Oncol..

[2] Pui C.-H., Mullighan C.G., Evans W.E., Relling M.V. (2012). Pediatric acute lymphoblastic leukemia: Where are we going and how do we get there?. Blood.

[3] Oskarsson T., Soderhall S., Arvidson J., Forestier E., Montgomery S., Bottai M., Lausen B., Carlsen N., Hellebostad M., Lähteenmäki P. (2015). Relapsed childhood acute lymphoblastic leukemia in the Nordic countries: Prognostic factors, treatment and outcome. Haematologica.

[4] Rheingold S.R., Ji L., Xu X., Devidas M., Brown P.A., Gore L., Winick N.J., Carroll W.L., Hunger S., Raetz E.A. (2019). Prognostic factors for survival after relapsed acute lymphoblastic leukemia (ALL): A Children’s Oncology Group (COG) study. J. Clin. Oncol..

[5] Dijoseph J., Armellino D., Boghaert E., Khandke K., Dougher M., Sridharan L., Kunz A., Hamann P.R., Gorovits B., Udata C. (2004). Antibody-targeted chemotherapy with CMC-544: A CD22-targeted immunoconjugate of calicheamicin for the treatment of B-lymphoid malignancies. Blood.

[6] Food and Drug Administration (FDA) Besponsa. Summary of Product Characteristics. https://www.accessdata.fda.gov/drugsatfda_docs/label/2024/761040s003lbl.pdf.

[7] Balwierz A.I. A Randomized Trial of the I-BFM-SG for the Management of Childhood Non-B Acute Lymphoblastic Leukemia. Final Version of Therapy Protocol from 14 August 2009. http://www.bialaczka.org/wp-content/uploads/2016/10/ALLIC_BFM_2009.pdf.

[8] ALL-IC REL 2016 Version 1.1. https://semmelweis.hu/gyermekklinika2/files/2022/05/ALL-IC-REL-2016_v1.1_2019-09-16.pdf.

[9] Locatelli F., Zugmaier G., Mergen N., Bader P., Jeha S., Schlegel P.G., Bourquin J.P., Handgretinger R., Brethon B., Rössig C. (2022). Blinatumomab in pediatric relapsed/refractory B-cell acute lymphoblastic leukemia: RIALTO expanded access study final analysis. Blood Adv..

[10] Locatelli F., Zugmaier G., Rizzari C., Morris J.D., Gruhn B., Klingebiel T., Parasole R., Linderkamp C., Flotho C., Petit A. (2021). Effect of Blinatumomab vs Chemotherapy on Event-Free Survival Among Children with High-risk First-Relapse B-Cell Acute Lymphoblastic Leukemia: A Randomized Clinical Trial. JAMA.

[11] Brown P.A., Ji L., Xu X., Devidas M., Hogan L.E., Borowitz M.J., Raetz E.A., Zugmaier G., Sharon E., Bernhardt M.B. (2021). Effect of Postreinduction Therapy Consolidation with Blinatumomab vs Chemotherapy on Disease-Free Survival in Children, Adolescents, and Young Adults With First Relapse of B-Cell Acute Lymphoblastic Leukemia: A Randomized Clinical Trial. JAMA.

[12] Messinger Y.H., Gaynon P.S., Sposto R., van der Giessen J., Eckroth E., Malvar J., Bostrom B.C., Therapeutic Advances in Childhood Leukemia & Lymphoma (TACL) Consortium (2012). Bortezomib with chemotherapy is highly active in advanced B-precursor acute lymphoblastic leukemia: Therapeutic Advances in Childhood Leukemia & Lymphoma (TACL) Study. Blood.

[13] Bertaina A., Vinti L., Strocchio L., Gaspari S., Caruso R., Algeri M., Coletti V., Gurnari C., Romano M., Cefalo M.G. (2017). The combination of bortezomib with chemotherapy to treat relapsed/refractory acute lymphoblastic leukaemia of childhood. Br. J. Haematol..

[14] Kyriakidis I., Mantadakis E., Stiakaki E., Groll A.H., Tragiannidis A. (2022). Infectious Complications of Targeted Therapies in Children with Leukemias and Lymphomas. Cancers.

[15] Raetz E.A., Cairo M.S., Borowitz M.J., Blaney S.M., Krailo M.D., Leil T.A., Reid J.M., Goldenberg D.M., Wegener W.A., Carroll W.L. (2008). Chemoimmunotherapy reinduction with epratuzumab in children with acute lymphoblastic leukemia in marrow relapse: A Children’s Oncology Group Pilot Study. J. Clin. Oncol..

[16] Shah N.N., Stevenson M.S., Yuan C.M., Richards K., Delbrook C., Kreitman R.J., Pastan I., Wayne A.S. (2015). Characterization of CD22 expression in acute lymphoblastic leukemia. Pediatr. Blood Cancer.

[17] Kantarjian H.M., DeAngelo D.J., Stelljes M., Liedtke M., Stock W., Gökbuget N., O’Brien S.M., Jabbour E., Wang T., Liang White J. (2019). Inotuzumab ozogamicin versus standard of care in relapsed or refractory acute lymphoblastic leukemia: Final report and long-term survival follow-up from the randomized, phase 3 INO-VATE study. Cancer.

[18] Bhojwani D., Sposto R., Shah N.N., Rodriguez V., Yuan C., Stetler-Stevenson M., O’Brien M.M., McNeer J.L., Quereshi A., Cabannes A. (2019). Inotuzumab ozogamicin in pediatric patients with relapsed/refractory acute lymphoblastic leukemia. Leukemia.

[19] Calvo C., Cabannes-Hamy A., Adjaoud D., Bruno B., Blanc L., Boissel N., Tabone M.D., Willson-Plat G., Villemonteix J., Baruchel A. (2020). Inotuzumab ozogamicin compassionate use for French paediatric patients with relapsed or refractory CD22-positive B-cell acute lymphoblastic leukaemia. Br. J. Haematol..

[20] Brivio E., Locatelli F., Lopez-Yurda M., Malone A., Díaz-de-Heredia C., Bielorai B., Rossig C., van der Velden V.H.J., Ammerlaan A.C.J., Thano A. (2021). A phase 1 study of inotuzumab ozogamicin in pediatric relapsed/refractory acute lymphoblastic leukemia (ITCC-059 study). Blood.

[21] Nakayama H., Ogawa C., Sekimizu M., Fujisaki H., Kosaka Y., Hashimoto H., Saito A.M., Horibe K. (2022). A phase I study of inotuzumab ozogamicin as a single agent in pediatric patients in Japan with relapsed/refractory CD22-positive acute lymphoblastic leukemia (INO-Ped-ALL-1). Int. J. Hematol..

[22] O’Brien M.M., Ji L., Shah N.N., Rheingold S.R., Bhojwani D., Yuan C.M., Xu X., Yi J.S., Harris A.C., Brown P.A. (2022). Phase II Trial of Inotuzumab Ozogamicin in Children and Adolescents with Relapsed or Refractory B-Cell Acute Lymphoblastic Leukemia: Children’s Oncology Group Protocol AALL1621. J. Clin. Oncol..

[23] Pennesi E., Michels N., Brivio E., van der Velden V.H.J., Jiang Y., Thano A., Ammerlaan A.J.C., Boer J.M., Beverloo H.B., Sleight B. (2022). Inotuzumab ozogamicin as single agent in pediatric patients with relapsed and refractory acute lymphoblastic leukemia: Results from a phase II trial. Leukemia.

